# Baseline cardiometabolic profiles and SARS-CoV-2 infection in the UK Biobank

**DOI:** 10.1371/journal.pone.0248602

**Published:** 2021-04-01

**Authors:** Ryan J. Scalsky, Yi-Ju Chen, Karan Desai, Jeffery R. O’Connell, James A. Perry, Charles C. Hong

**Affiliations:** 1 Medical Scientist Training Program, University of Maryland School of Medicine, Baltimore, Maryland, United States of America; 2 Department of Medicine, University of Maryland School of Medicine, Baltimore, Maryland, United States of America; University of Milano, ITALY

## Abstract

**Background:**

SARS-CoV-2 is a rapidly spreading coronavirus responsible for the Covid-19 pandemic, which is characterized by severe respiratory infection. Many factors have been identified as risk factors for SARS-CoV-2, with much early attention being paid to body mass index (BMI), which is a well-known cardiometabolic risk factor.

**Objective:**

This study seeks to examine the impact of additional baseline cardiometabolic risk factors including high density lipoprotein-cholesterol (HDL-C), low density lipoprotein-cholesterol (LDL-C), Apolipoprotein A-I (ApoA-I), Apolipoprotein B (ApoB), triglycerides, hemoglobin A1c (HbA1c) and diabetes on the odds of testing positive for SARS-CoV-2 in UK Biobank (UKB) study participants.

**Methods:**

We examined the effect of BMI, lipid profiles, diabetes and alcohol intake on the odds of testing positive for SARS-Cov-2 among 9,005 UKB participants tested for SARS-CoV-2 from March 16 through July 14, 2020. Odds ratios and 95% confidence intervals were computed using logistic regression adjusted for age, sex and ancestry.

**Results:**

Higher BMI, Type II diabetes and HbA1c were associated with increased SARS-CoV-2 odds (p < 0.05) while HDL-C and ApoA-I were associated with decreased odds (p < 0.001). Though the effect of BMI, Type II diabetes and HbA1c were eliminated when HDL-C was controlled, the effect of HDL-C remained significant when BMI was controlled for. LDL-C, ApoB and triglyceride levels were not found to be significantly associated with increased odds.

**Conclusion:**

Elevated HDL-C and ApoA-I levels were associated with reduced odds of testing positive for SARS-CoV-2, while higher BMI, type II diabetes and HbA1c were associated with increased odds. The effects of BMI, type II diabetes and HbA1c levels were no longer significant after controlling for HDL-C, suggesting that these effects may be mediated in part through regulation of HDL-C levels. In summary, our study suggests that baseline HDL-C level may be useful for stratifying SARS-CoV-2 infection risk and corroborates the emerging picture that HDL-C may confer protection against sepsis in general and SARS-CoV-2 in particular.

## Introduction

Since early December 2019, when the first cases of the severe acute respiratory syndrome coronavirus 2 (SARS-CoV-2 or Covid-19) were identified in Wuhan, China, nearly 13 million individuals have tested positive for the virus [1]. Researchers have rapidly attempted to define the clinical characteristics associated with increased risk of becoming infected with SARS-CoV-2 to improve our understanding and clinical management of this pandemic. Early data from across the globe have identified pre-existing cardiovascular disease and obesity as risk factors associated with acquiring SARS-CoV-2 [2–8]. Curiously, early data from China also found that hypolipidemia and declining HDL-C at the time of acute COVID19 illness was associated with disease severity [9, 10]. There remains limited research on how an individual’s baseline cardiometabolic profile, specifically lipid levels, affect one’s risk for contracting the virus. This has received particular attention as the known viral entry mechanism for SARS-CoV-1, a closely related virus responsible for the 2003 SARS outbreak in China, has been shown, in preliminary in-vitro studies, to be cholesterol-dependent [11]. In this paper we analyze the association of a positive SARS-CoV-2 test with an individual’s lipid profile in the UK Biobank resource.

## Methods

The UK Biobank resource began releasing SARS-CoV-2 test results in April 2020 to approved researchers. Data collected by the UK Biobank resource covers a wide variety of areas that researchers apply for access to. This data was not collected for this study but rather this study leveraged available collected data. This data was consented for by participants through UK Biobank protocol and was fully de-identified prior to approved researchers accessing it. Full details on these test results are available online [12]. Using test results released on July 14, 2020, we classified subjects testing positive for SARS-CoV-2 as cases. If multiple tests were performed, we classified a subject as a case if any test gave a positive result, based on the rationale that false positives are less likely than false negatives. Those with only negative test results were classified as controls. Tests were initially conducted in hospital settings in individuals who presented with respiratory symptoms. From April 27^th^ onward, testing was expanded to include community clinics and all non-elective patients admitted overnight, including those who were asymptomatic. 70.1% of the 9,005 subjects (cases and controls) were inpatient when the sample was taken, 74.1% of 7,497 controls were inpatient when the sample was taken and 50.3% of 1,508 cases were inpatient when the sample was taken. The vast majority of samples for testing were obtained by nose/throat swabs and samples were analyzed for SARS-CoV-2 RNA via PCR.

The association analysis was performed with Plink2 [13] using logistic regression. The binary outcome variable of “SARS-CoV-2 test status” (cases tested positive, controls tested negative) was run against a series of independent variables which included continuous, categorical, and binary ICD10 data supplied by the UK Biobank. The data for continuous and categorical traits were collected when subjects were enrolled into the UK Biobank (2006–2010). Serum was collected for analysis of LDL-C, HDL-C, ApoA-I, ApoB and triglycerides. LDL-C was analyzed by enzymatic selective protection, HDL-C was analyzed by enzyme immunoinhibition, ApoA-I and ApoB were analyzed by immunoturbidimetric analysis and triglycerides were measured by GPO-POD. All analyses were completed using the AU5800 by Beckman Coulter. Height and weight were measured, and BMI was computed from these values. Impedance BMI was measured by bioelectrical impedance using the Tanita BC418MA body composition analyzer. Additional details on collection and analysis of biomarkers (e.g. LDL-C, HDL-C, ApoA-I) are available from the UK Biobank website [14]. ICD10 diagnostic codes are current for all subjects through October 2019. We also grouped the ICD10 code data into Phecodes in order to increase statistical power [15]. The analysis included covariates of sex, age, and principal components (PCs) 1 through 4 to adjust for ancestry. Principal component analysis, a standard technique used in statistical genetics, generates a dataset of PCs (typically 10) that can be used as covariates to correct for population stratification (i.e. differences in ancestry) [16]. PCs provided by the UK Biobank, which were computed from the cohort’s genotypes, were used. Our preliminary analysis showed that only the first 4 PCs were significant at p < 0.05 and thus we included only PC1-4 as covariates.

Our analysis yielded odds ratios (OR) and 95% confidence intervals (CI) for each trait tested against the “SARS-CoV-2 test status”. An OR greater than 1.0 indicates increased odds of a SARS-CoV-2 positive test compared to the controls. An OR less than 1.0 indicates decreased odds. For continuous phenotypes, the OR indicates the increased odds (for OR > 1.0) or decreased odds (for OR < 1.0) per standard deviation increase in the continuous phenotype.

## Results

### Demographics

This dataset includes 1,508 cases and 7,497 controls for a total of 9,005 subjects. Prior to the association analysis we compared the cases and controls for differences in sex, ancestry and age. Significant differences were found between the sex (p-value = 1.3x10^-3^; Table 1), ancestry (p-value = 1.1x10^-15^; Table 1) and age (p-value = 3.4x10^-8^; Table 1) of cases and controls.

**Table 1:** Demographics—Sex, ancestry and age.

	N	Male (%)	Female (%)	White (%)	Non-white (%)	Age (SD)
Cases	1,508	796 (52.8)	712 (47.2)	1312 (87.0)	196 (13.0)	67.39 (9.22)
Controls	7,497	3,618 (48.3)	3,879 (51.7)	6980 (93.1)	517 (6.9)	68.81 (8.38)
All	9,005	4,414 (49.0)	4,591 (51.0)	8292 (92.1)	713 (7.9)	68.57 (8.54)
% positive	16.7%	18.0%	15.5%	15.8%	27.5%	N/A
p-value		1.3 x 10^−3^		1.1 x 10^−15^		3.4 x 10^−8^

*N/Male/Female/Non-white/White indicate number of subjects. Age is the mean age as of 2020, SD is standard deviation. P-values are from chi-squared test for sex and ancestry, and t-test for age, comparing cases and controls. White ancestry includes subjects self-reporting as White, British, Irish, or “Any other white background”. Non-white ancestry includes all other self-report categories.

### BMI

Body mass index (BMI) has been shown to increase SARS-CoV-2 risk across many populations [3]. Interestingly, we found that BMI was associated with an increased odds of SARS-CoV-2 positive testing (OR = 1.12, 95% CI = 1.06–1.18, p-value = 6.14x10^-5^; Table 2) but when HDL was controlled for this effect was no longer significant (OR = 1.06, 95% CI = 0.995–1.13, p-value = 0.071; Table 2). These findings were consistent when BMI was measured by electrical impedance (OR = 1.12, 95% CI = 1.06–1.18, p-value = 8.54x10^-5^; Table 2) and the significance was also lost when HDL-C was controlled for (OR = 1.06, 95% CI = 0.994–1.13, p-value = 0.077; Table 2).

**Table 2:** Effect of body mass index.

Trait	Covariates	N	Cases/Controls	Mean (S.D.)	Odds Ratio	95% Confidence Interval	p-value
BMI	Age, Sex, 4pc	8,939	1,496/7,443	28.29 (5.27)	1.12	1.06–1.18	**6.14x10**^**-5**^
BMI	Age, Sex, 4pc, HDL-C	7,764	1,280/6,484	28.31 (5.26)	1.06	0.995–1.13	0.071
Impedance BMI	Age, Sex, 4pc	8,739	1,463/7,276	28.29 (5.26)	1.12	1.06–1.18	**8.54x10**^**-5**^
Impedance BMI	Age, Sex, 4pc, HDL-C	7,590	1,252/6,338	28.30 (5.25)	1.06	0.994–1.13	0.077

*Body Mass Index (BMI) measured by height and weight in units of Kg/m^2^, Impedance BMI measured in increments of 0.1 in units of Kg/m^2^, 4pc (4 principle components to account for ancestry).

### HDL-cholesterol/ApoA-I

After controlling for age, sex and 4pc, we found that plasma HDL-C levels were associated with a reduced odds of testing positive for SARS-CoV-2 (OR = 0.845, 95% CI = 0.788–0.907, p-value = 2.45x10^-6^; Table 3), this effect was maintained even when controlling for BMI (OR = 0.863, 95% CI = 0.801–0.93, p-value = 1.17x10^-4^; Table 3). Moreover, we found that plasma levels of Apolipoprotein A-I (ApoA-I), the major protein component of HDL-C particles in plasma, were also associated with a reduced odds of testing positive for SARS-CoV-2 (OR = 0.849, 95% CI = 0.793–0.910, p-value = 2.90x10^-6^; Table 3). The effect of ApoA-I also remained significant when controlling for BMI (OR = 0.865, 95% CI = 0.806–0.929, p-value = 6.97x10^-5^; Table 3). Consistent with high collinearity between HDL-C and ApoA-I, the effect of either is negligible when the opposite is controlled for suggesting that both describe the same effect (Table 3).

**Table 3:** Effect of HDL and ApoA.

Trait	Covariates	N	Cases/Controls	Mean (S.D.)	Odds Ratio	95% Confidence Interval	p-value
HDL-C	Age, Sex, 4pc	7,821	1,291/6,530	1.40 (0.38)	0.845	0.788–0.907	**2.45x10**^**-6**^
	Age, Sex, 4pc, ApoA-I	7,777	1,286/6,491	1.39 (0.37)	0.944	0.804–1.11	0.480
	Age, Sex, 4pc, BMI	7,764	1,280/6,484	1.40 (0.38)	0.863	0.801–0.93	**1.17x10**^**-4**^
ApoA-I	Age, Sex, 4pc	7,783	1,288/6,495	1.51 (0.27)	0.849	0.793–0.910	**2.90x10**^**-6**^
	Age, Sex, 4pc, HDL-C	7,777	1,286/6,491	1.51 (0.27)	0.894	0.762–1.048	0.167
	Age, Sex, 4pc, BMI	7,727	1,277/6,450	1.51 (0.27)	0.865	0.806–0.929	**6.97x10**^**-5**^

*High density lipoprotein (HDL-C) measured in mmol/L, Apolipoprotein A-I (ApoA-I) measured in g/L.

### Hyperlipidemia/LDL-cholesterol/ApoB/triglycerides

The diagnosis of hyperlipidemia (ICD10 codes E78.4 and E78.5) was modestly associated with an elevated odds of testing positive for SARS-CoV-2 (OR = 1.362, 95% CI = 1.021–1.817, p-value 0.036; Table 4). However, when ApoA-I, HDL-C or BMI were controlled for, this effect was no longer significant (Table 4). In contrast to prior studies, we did not find any association between LDL-C levels and odds of testing positive for SARS-CoV-2 (OR = 0.995, 95% CI = 0.939–1.055, p-value = 0.872; Table 4) [9]. Consistent with this, apolipoprotein B, the primary lipoprotein associated with plasma LDL-C, was not associated with any significant effect on odds of testing positive for SARS-CoV-2 (OR = 1.003, 95% CI = 0.947–1.063, p-value = 0.910; Table 4). Additionally, no significant effect of triglyceride levels on odds of testing positive for SARS-CoV-2 were found (OR = 1.026, 95% CI = 0.969–1.087, p-value = 0.375; Table 4).

**Table 4:** Effect of hyperlipidemia, LDL, ApoB and triglycerides.

Trait	Covariates	N	Cases/Controls	Mean (S.D.)	Odds Ratio	95% Confidence Interval	p-value
Hyperlipidemia	Age, Sex, 4pc	7,515	1,277/6,238	N/A	1.351	1.010–1.807	**0.043**
	Age, Sex, 4pc, HDL-C	6,522	1,083/5,439	N/A	1.261	0.916–1.736	0.155
	Age, Sex, 4pc, ApoA-I	6,489	1,081/5,408	N/A	1.273	0.924–1.753	0.140
	Age, Sex, 4pc, BMI	5,957	1,267/6,195	N/A	1.259	0.937–1.69	0.126
LDL-C	Age, Sex, 4pc	8,532	1,413/7,119	3.44 (0.89)	0.995	0.939–1.055	0.872
ApoB	Age, Sex, 4pc	8,498	1,404/7,094	1.01 (0.24)	1.003	0.947–1.063	0.910
Triglycerides	Age, Sex, 4pc	8,534	1,411/7,123	1.81 (1.06)	1.026	0.969–1.087	0.375

*Low density lipoprotein (LDL-C) measured in mmol/L, Apolipoprotein B (ApoB) measured in g/L, triglycerides measured in mmol/L.

### Diabetes/HbA1c

The diagnosis of Type II diabetes (Phecode phe250.2) was associated with an elevated odds of testing positive for SARS-CoV-2 (OR = 1.213, 95% CI = 1.028–1.432, p-value = 0.0225; Table 5). Consistent with this, we found that HbA1c level was associated with a significant increase in odds of testing positive for SARS-CoV-2 (OR = 1.061, 95% CI = 1.005–1.121, p-value = 0.0332; Table 5). However, when ApoA-I, HDL-C or BMI were controlled for, the effects of both the type II diabetes diagnosis and HgA1c level were no longer significant (Table 5). Curiously, Type I diabetes (Phecode phe250.1) was not associated with an elevated odds of testing positive for SARS-CoV-2 (Table 5).

**Table 5:** Effect of diabetes and HbA1c.

Trait	Covariates	N	Cases/Controls	Mean (S.D.)	Odds Ratio	95% Confidence Interval	p-value
Type II Diabetes	Age, Sex, 4pc	8,948	1,500/7,448	N/A	1.213	1.028–1.432	**0.023**
	Age, Sex, 4pc, HDL-C	7,770	1,283/6,487	N/A	1.131	0.943–1.356	0.184
	Age, Sex, 4pc, BMI	8,882	1,488/7,394	N/A	1.126	0.947–1.34	0.180
HbA1c*	Age, Sex, 4pc	8,508	1,421/7,087	37.32 (8.43)	1.061	1.005–1.121	**0.033**
	Age, Sex, 4pc, HDL-C	7,398	1,217/6,181	37.39 (8.51)	1.035	0.974–1.100	0.265
	Age, Sex, 4pc, BMI	8,444	1,409/7,035	37.30 (8.41)	1.03	0.972–1.09	0.317
Type I Diabetes	Age, Sex, 4pc	8,006	1,311/6,695	N/A	0.817	0.529–1.261	0.360

*HbA1c measured in mmol/mol.

## Discussion

Early studies of SARS-CoV-2 pandemic identified pre-existing cardiovascular disease and obesity as risk factors associated with acquiring SARS-CoV-2 [2–8]. In addition prior studies found that hypolipidemia and declining HDL-C in the setting of acute Covid-19 illness was associated with disease severity [9, 10]. Moreover, obesity was associated with higher prevalence of SARS-CoV-2 infection and Covid-19 disease severity [3, 7, 8]. Here, we sought to validate these findings by examining the potential effects of baseline BMI, lipoproteins, their respective apolipoproteins and diabetes on the odds of acquiring SARS-CoV-2 in a cohort from the UK Biobank dataset. We confirmed that baseline hyperlipidemia was associated with the odds of testing positive for SARS-CoV-2, but this association was driven primarily by association of baseline lower HDL-C and ApoA-I levels with SARS-CoV-2 positivity, suggesting specifically that baseline HDL-C level may be useful for stratifying SARS-CoV-2 infection risk.

Moreover, consistent with an earlier study, we confirmed the association of high BMI with the odds of testing positive for SARS-CoV-2 [7]. Importantly, this effect was no longer significant when baseline HDL-C and ApoA-I levels were controlled for. We also found association of Type II (but not Type I) Diabetes and HbA1c levels with SARS-CoV-2 diagnosis. Again, when baseline ApoA-I or HDL-C levels were controlled for, the effects of both the Type II diabetes diagnosis and HbA1c level were no longer significant. Given that Type II diabetes is associated with reduced HDL-C levels, we hypothesize that the elevated odds associated with Type II diabetes is mediated in part through its effect on HDL-C levels. Taken together, our results suggest that HDL-C may be mediating part of the well-known effect of BMI on SARS-CoV-2 /Covid-19 risk and plays a greater role in SARS-CoV-2 pathogenesis than previously appreciated.

Although the findings of this study persist when appropriate controls are applied, we acknowledge the inherent limitations of this association study, which is subject to sampling bias. Importantly, we do not know the context in which the SARS-CoV-2 testing was carried out, the HDL-C status at the time of testing, and the disease severity of each case. Participants tested in this study were primarily those who presented to a clinical care site with symptoms suggestive of SARS-CoV-2. Although this has the potential to marginally increase SARS-CoV-2 positive testing rate, it is unlikely to influence the association of HDL-C, BMI and alcohol consumption with SARS-CoV-2 positivity rate. Additionally, while majority of those tested were inpatient at the time of sampling, we acknowledge the potential confounding effects of subsequent expansion of testing into the community and to asymptomatic patients; however, we believe that such effects would tend to diminish any association with SARS-CoV-2 test positive rates.

Another limitation of an association study based on UK Biobank is that the baseline cardiometabolic data was collected several years prior to the SARS-CoV-2 pandemic. However, we do not believe that this did not have a substantial impact on our findings. While mean BMI has been reported to have increased around the world from 1976 to 2016, this increase has plateaued in recent years in high-income English-speaking countries, including the UK [17]. Additionally, mean triglyceride, HDL-C and LDL-C levels tend to change only modestly or remain relatively stable in the population over time [18, 19]. We believe that these modest changes over time would tend to diminish their associations with SARS-CoV-2 test positive rates, particularly if abnormal baseline levels were treated in the interim with medications. While the concurrent use of medications at the time of SARS-CoV-2 infection among the UKB participants is unknown, we examined the use of common cardiometabolic medications at the time of enrollment and found no association with the SARS-CoV-2 infection, except for metformin, whose significance disappeared when adjusted for HDL-C levels or BMI (S1 Data). Too few subjects (<1% of UKB participants) were on either niacin or a cholesteryl ester transfer protein (CEPT) inhibitor, which are known to raise HDL-C levels, to determine association with SARS-CoV-2 infection.

Despite these limitations, we believe that our exploration of SARS-CoV-2 corroborates some of the earlier studies and provides valuable insight and guidance for future studies. One of the most compelling finding of this study is the association of lower baseline HDL-C levels with SARS-CoV-2 positivity, which corroborates an earlier study, which found association of declining HDL-C levels with Covid-19 disease severity [10]. While causal inferences are beyond the scope of this study, and the potential mechanism by which HDL-C may confer protection from SARS-CoV-2 is unknown, given HDL-C’s pleiotropic characteristics including antioxidant, antithrombotic, microvascular-protective, anti-apoptotic, and anti- as well as pro-inflammatory properties, it is plausible that HDL-C may play a protective role in preventing the establishment of SARS-CoV-2 infection [20–22]. Indeed, based on the protective effects of HDL-C/ApoA-I replacement strategies and cholesteryl ester transfer protein (CEPT) inhibition in septic shock, there is increasing recognition of the role of HDL-C in infection control, including the direct antiviral effects of HDL-C [21–24]. Our analysis opens up several avenues for further study, for example, to determine whether baseline HDL-C levels can identify high risk patients, to explore whether administration of CEPT inhibitors such as ezetimibe to elevate HDL-C levels may confer protection against SARS-CoV-2 infection, or even to examine whether HDL-C particle confers direct protection against SARS-CoV-2 infection.

## Supporting information

S1 Data (XLSX)

## References

[1] Berry, I., Brewer, T., & Brownstein, J. (2020). COVID-19. Retrieved November 11, 2020, from https://www.healthmap.org/covid-19/

[2] Simonnet A, Chetboun M, Poissy J, et al. High prevalence of obesity in severe acute respiratory syndrome coronavirus-2 (SARS-CoV-2) requiring invasive mechanical ventilation. Obesity. 4 2020. 10.1002/oby.22831 32271993 PMC7262326

[3] Lighter Jennifer, et al. “Obesity in Patients Younger Than 60 Years Is a Risk Factor for COVID-19 Hospital Admission.” *Clinical Infectious Diseases*, vol. 71, no. 15, 2020, pp. 896–897., 10.1093/cid/ciaa415 32271368 PMC7184372

[4] Wu Z, McGoogan JM. Characteristics of and Important Lessons from the Coronavirus Disease 2019 (COVID-19) Outbreak in China: Summary of a Report of 72314 Cases from the Chinese Center for Disease Control and Prevention. JAMA—J Am Med Assoc. 2020;323(13):1239–1242. 10.1001/jama.2020.2648 32091533

[5] Richardson S, Hirsch JS, Narasimhan M, et al. Presenting Characteristics, Comorbidities, and Outcomes Among 5700 Patients Hospitalized With COVID-19 in the New York City Area. *JAMA*. 2020;323(20):2052–2059. 10.1001/jama.2020.6775 32320003 PMC7177629

[6] Goyal P, Choi JJ, Pinheiro LC, et al. Clinical Characteristics of Covid-19 in New York City. *N Engl J Med*. 2020;382(24):2372–2374. 10.1056/NEJMc2010419 32302078 PMC7182018

[7] Kassir R. (2020). Risk of COVID-19 for patients with obesity. *Obesity Reviews*, 21(6). 10.1111/obr.13034 32281287 PMC7235532

[8] Popkin B. M., Du S., Green W. D., Beck M. A., Algaith T., Herbst C. H., et al. (2020). Individuals with obesity and COVID-19: A global perspective on the epidemiology and biological relationships. *Obesity Reviews*. 10.1111/obr.13128 32845580 PMC7461480

[9] Wei X., Zeng W., Su J., Wan H., Yu X., Cao X., et al. (2020). Hypolipidemia is associated with the severity of COVID-19. *Journal of Clinical Lipidology*, 14(3), 297–304. 10.1016/j.jacl.2020.04.008 32430154 PMC7192140

[10] Hu X., Chen D., Wu L., He G., & Ye W. (2020). Declined serum high density lipoprotein cholesterol is associated with the severity of COVID-19 infection. Clinica Chimica Acta, 510, 105–110. 10.1016/j.cca.2020.07.015 32653486 PMC7350883

[11] Lu Y., Liu D. X. & Tam J. P. Lipid rafts are involved in SARS-CoV entry into Vero E6 cells. Biochem. Biophys. Res. Commun. 369, 344–349 (2008) 10.1016/j.bbrc.2008.02.023 18279660 PMC7092920

[12] Current and future availability of COVID-19 related health outcome data. (n.d.). Retrieved July 22, 2020, from http://biobank.ndph.ox.ac.uk/ukb/exinfo.cgi?src=COVID19_availability

[13] Chang CC, Chow CC, Tellier LC, Vattikuti S, Purcell SM, Lee JJ. Second-generation PLINK: rising to the challenge of larger and richer datasets. Gigascience. 2015;4:7. Published 2015 Feb 25. 10.1186/s13742-015-0047-8 25722852 PMC4342193

[14] Biomarkers: UK Biobank. (2019). Retrieved November 11, 2020, from https://www.ukbiobank.ac.uk/uk-biobank-biomarker-panel/

[15] Wu P, Gifford A, Meng X, et al. Mapping ICD-10 and ICD-10-CM Codes to Phecodes: Workflow Development and Initial Evaluation. JMIR Med Inform. 2019;7(4):e14325. Published 2019 Nov 29. 10.2196/14325 31553307 PMC6911227

[16] Price A. J., Patterson N. J., Plenge R. M., Weinblatt M. E., Shadick N. A., & Reich D. (2006). Principal components analysis corrects for stratification in genome-wide association studies. *Nature Genetics*, 38, 904–909. Retrieved September 29, 2020, from https://www.nature.com/articles/ng1847 10.1038/ng1847 16862161

[17] Bentham James, et al. “Worldwide Trends in Body-Mass Index, Underweight, Overweight, and Obesity from 1975 to 2016: a Pooled Analysis of 2416 Population-Based Measurement Studies in 128·9 Million Children, Adolescents, and Adults.” *The Lancet*, 10 10. 2017, 10.1016/S0140-6736(17)32129-3 PMC5735219 29029897

[18] Ding Wenqing, et al. “10-Year Trends in Serum Lipid Levels and Dyslipidemia Among Children and Adolescents From Several Schools in Beijing, China.” *Journal of Epidemiology*, vol. 26, no. 12, 9 7 2016, pp. 637–645., 10.2188/jea.JE20140252 27397598 PMC5121432

[19] Tian Jing, et al. “Trends in the Levels of Serum Lipids and Lipoproteins and the Prevalence of Dyslipidemia in Adults with Newly Diagnosed Type 2 Diabetes in the Southwest Chinese Han Population during 2003–2012.” *International Journal of Endocrinology*, vol. 2015, 18 5 2015, pp. 1–7., 10.1155/2015/818075 26089896 PMC4451154

[20] White R., Giordano S., & Datta G. (2017). Role of HDL-Associated Proteins and Lipids in the Regulation of Inflammation. *Advances in Lipoprotein Research*. 10.5772/67141

[21] Tanaka S., Couret D., Tran-Dinh A., Duranteau J., Montravers P., Schwendeman A., et al. (2020). High-density lipoproteins during sepsis: From bench to bedside. *Critical Care*, 24(1). 10.1186/s13054-020-02860-3 32264946 PMC7140566

[22] Blauw L. L., Wang Y., Dijk K. W., & Rensen P. C. (2020). A Novel Role for CETP as Immunological Gatekeeper: Raising HDL to Cure Sepsis? *Trends in Endocrinology & Metabolism*, 31(5), 334–343. 10.1016/j.tem.2020.01.003 32033866

[23] Trinder M., Wang Y., Madsen C. M., Ponomarev T., Bohunek L., Daisley B. A., et al. (2020). Inhibition of Cholesteryl Ester Transfer Protein Preserves High-Density Lipoprotein Cholesterol and Improves Survival in Sepsis. *Circulation*. 10.1161/CIRCULATIONAHA.120.048568 33228395

[24] Singh I. P., Chopra A. K., Coppenhaver D. H., Ananatharamaiah G., & Baron S. (1999). Lipoproteins account for part of the broad non-specific antiviral activity of human serum. *Antiviral Research*, 42(3), 211–218. 10.1016/s0166-3542(99)00032-7 10443533

