# Supplementary material for: Baseline cardiometabolic profiles and SARS-CoV-2 infection in the UK Biobank
Source: PLoS One. 2021 Apr 1;16(4):e0248602. doi: 10.1371/journal.pone.0248602 (PMC8016301; doi:10.1371/journal.pone.0248602)
Supplement: Attachment — Submitted filename: Rebuttal Letter 1-15-2021.doc [file pone.0248602.s002.doc]

We thank the Reviewers for their expert comments on our original submission, and have made the appropriate revisions. Our response to specific comments are provided below.

**Reviewer #1:**

- Please refer to HDL and LDL as HDL-cholesterol and LDL-cholesterol, which are more appropriate since you are measuring cholesterol and not the particles themselves.

*We have made the corrections as requested.*

- Please refer to apoA as apoA-I, since different apoA exist, with different biological roles.

*We have made the corrections as requested.*

- Check abbreviations. They should be defined the first time they appear.

*We have made the corrections as requested.*

- It would be useful to include a more detailed characterization of the included patients. Since lipids and other biochemical parameter were collected at the time of enrolment in the database, lipid-lowering treatment and hypoglycemic medications should be included in the description of patients and in the analysis.

*We have included the analysis of the impact of lipid-lowering treatment and diabetes medications at the time of enrollment as Supplementary Data and dissuss them in the discussion. Briefly, while the concurrent use of medications at the time of SARS-CoV-2 infection among the UKB participants is unknown, we examined the use of common cardiometabolic medications at the time of enrollment and found no association with the SARS-CoV-2 infection, except for metformin, whose significance disappeared when adjusted for HDL-C levels or BMI.*

- If available, association of these parameters with disease severity would be of interest.

*Disease severity data is not available from the UK Biobank.*

**Reviewer #2**

- Besides pharmacological approaches, the outbreak COVID-19 has led the scientific community to find biomarker that could have help to identify people who are at the risk the most. Although the present article is aimed at identifying which metabolic biomarker could be more suitable to identify these individuals, the manuscript in this form seems to fail in some statements, e.g., which is the portrait linking red wine and COVID-19? More specifically, which is the scientific path bonding drinking intake and all the other parameters? I would leave out this parameter. Instead, which is the role of insulin? We agree with the Reviewer 2; therefore, we have removed the section on red wine intake.

We examine the impact of insulin on *SARS-CoV-2 infection and found no association. This information* is now included in the Data Supplement.

- The aims should be described in a more detailed way.

*We have modified the objective in the abstract to specifically state that “this study seeks to examine the impact of additional baseline cardiometabolic risk factors including high density lipoprotein-cholesterol (HDL-C), low density lipoprotein-cholesterol (LDL-C), Apolipoprotein A-I (ApoA-I), Apolipoprotein B (ApoB), triglycerides, hemoglobin A1c (HbA1c) and diabetes on the odds of testing positive for SARS-CoV-2 in UK Biobank (UKB) study participants.”*

- Overall, as stated in the limitations, these parameters have been collected several years prior the outbreak and thus no recent records exist. Lipoproteins are closely related to infections and recent data on COVID-19 have correlated lipoproteins with outcomes (death) instead of odds of testing positive for SARS-CoV-2. Which is a plausible explanation? The discussion should address mechanist pathways related to the parameters that have been analyzed.

*We agree with the reviewer #2 and have expanded discourse on these topics in the Discussions. To paraphrase, while the present study only presents associations between HDL-C and SARS-CoV-2 infectivity, our findings corroborate an earlier study, which found association of declining HDL-C levels with sepsis in general and Covid-19 disease severity in particular. Together with the emerging data on the protective effects of HDL-C/ApoA-I replacement strategies and cholesteryl ester transfer protein (CEPT) inhibition in septic shock, and increasing recognition of the role of HDL-C in infection control, including the direct antiviral effects of HDL-C, we believe that our study highlights mechanistic and therapeutic insights that deserves further attention.*
